# Bis[1-benzyl-3-(4-methyl­phen­yl)imidazol-2-yl­idene]silver(I) hexa­fluorido­phosphate

**DOI:** 10.1107/S1600536811026675

**Published:** 2011-07-23

**Authors:** Kun Huang, Da-Bin Qin

**Affiliations:** a Department of Chemistry and Chemical Engineering, Sichuan University of Arts and Science, Dazhou 635000, People’s Republic of China; b School of Chemistry and Chemical Engineering, China West Normal University, Nanchong 637002, People’s Republic of China

## Abstract

The title silver *N*-heterocyclic carbene compound, [Ag(C_17_H_16_N_2_)_2_]PF_6_, crystallizes as a mononuclear salt. The two imidazole rings, which are almost coplanar [maximum deviation from the least squares plane of 0.05 (2) Å], are linked by the Ag atom with a C—Ag—C angle of 178.60 (9)°. In the crystal, C—H⋯F hydrogen bonds, weak π–π inter­actions [centroid–centroid distances = 3.921 (1) and 3.813 (3) Å] and C—H⋯π inter­actions lead to a supermolecular structure.

## Related literature

For the first silver *N*-heterocyclic carbene, see: Arduengo *et al.* (1993 ▶). For the role of *N*-heterocyclic carbene ligands in organometallic chemistry, see: Lin *et al.* (2009 ▶). For applications of silver *N*-heterocyclic carbenes, see: Nebioglu *et al.* (2007) ▶; Samantaray *et al.* (2007 ▶). For Ag—C bond lengths, see: Wang, Xu *et al.* (2005) ▶. For the synthesis of the title compound, see: Liu *et al.* (2003 ▶); Wang, Song *et al.* (2005 ▶). For a related structure, see: Catalano & Etogo (2007 ▶).
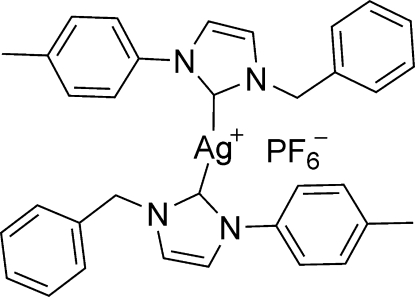


## Experimental

### 

#### Crystal data

[Ag(C_17_H_16_N_2_)_2_]PF_6_
*M* *_r_* = 749.48
Monoclinic, 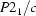

*a* = 9.692 (2) Å
*b* = 16.312 (4) Å
*c* = 20.227 (5) Å
β = 93.469 (3)°
*V* = 3192.1 (12) Å^3^
*Z* = 4
Mo *K*α radiation
μ = 0.75 mm^−1^
*T* = 113 K
0.20 × 0.18 × 0.12 mm

#### Data collection

Rigaku Saturn CCD area detector diffractometer
Absorption correction: multi-scan (*CrystalClear*; Rigaku/MSC, 2004 ▶) *T*_min_ = 0.865, *T*_max_ = 0.916
21677 measured reflections
5636 independent reflections
4775 reflections with *I* > 2σ(*I*)
*R*_int_ = 0.043

#### Refinement

*R*[*F* ^2^ > 2σ(*F* ^2^)] = 0.031
*wR*(*F* ^2^) = 0.081
*S* = 1.01
5636 reflections
417 parameters
H-atom parameters constrained
Δρ_max_ = 1.29 e Å^−3^
Δρ_min_ = −0.38 e Å^−3^

### 

Data collection: *CrystalClear* (Rigaku/MSC, 2004 ▶); cell refinement: *CrystalClear*; data reduction: *CrystalClear*; program(s) used to solve structure: *SHELXS97* (Sheldrick, 2008 ▶); program(s) used to refine structure: *SHELXL97* (Sheldrick, 2008 ▶); molecular graphics: *SHELXTL* (Sheldrick, 2008 ▶); software used to prepare material for publication: *CrystalStructure* (Rigaku/MSC, 2004 ▶).

## Supplementary Material

Crystal structure: contains datablock(s) global, I. DOI: 10.1107/S1600536811026675/qm2012sup1.cif

Structure factors: contains datablock(s) I. DOI: 10.1107/S1600536811026675/qm2012Isup2.hkl

Additional supplementary materials:  crystallographic information; 3D view; checkCIF report

## Figures and Tables

**Table 1:** Hydrogen-bond geometry (Å, °) *Cg*3 is the centroid of the C1–C6 ring.

*D*—H⋯*A*	*D*—H	H⋯*A*	*D*⋯*A*	*D*—H⋯*A*
C9—H9⋯F1^i^	0.95	2.54	3.120 (6)	119
C26—H26⋯F5^ii^	0.95	2.40	3.222 (1)	144
C34—H34*A*⋯F1^iii^	0.98	2.53	3.276 (8)	133
C27—H27⋯*Cg*3^ii^	0.95	2.50	3.295 (1)	140

Symmetry codes: (i) 
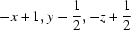
; (ii) 
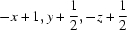
; (iii) 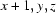.

**Fig. 1.**
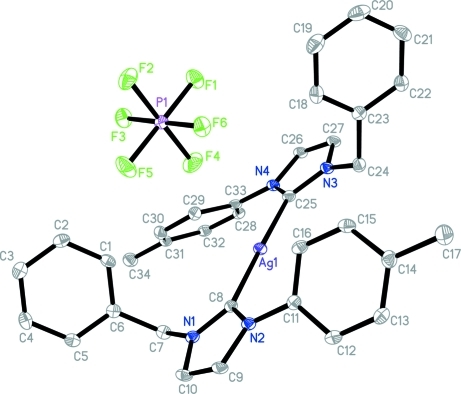
A view of the molecular structure of the title compound. The displacement ellipsoids are drawn at 30% probability level. C-bound H atoms have been omitted For clarity.

**Fig. 2.**
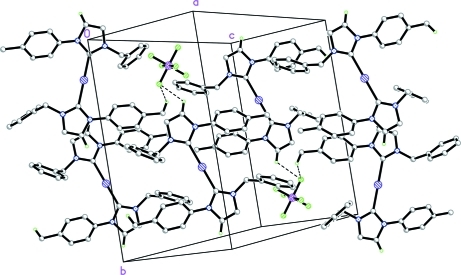
Crystal packing of the title compound, view down the a axis, showing the cations and anions linked via C—H···F interactions (dashed lines).H-atoms not involved in these interactions have been omitted for clarity.

## supplementary crystallographic information

### Comment

The discivery of first silver N-heterocyclic carbene (NHC) in 1993 by Arduengo (Arduengo *et al.*, 1993) led to its rapid use in organometallic chemistry (Lin *et al.*, 2009). silver NHCs can be used in various fields such as medical chemistry, Catalysis *et al.* (Nebioglu *et al.*, 2007; Samantaray *et al.*, 2007). Herein we report the crystal structure of the title silver NHC compound.

In the title compound, the silver(I) atom lies on a non-crystallographic twofold axis. The Ag—C bond lengths are close to literature values (Wang, Xu *et al.*, 2005). The silver coordination geometry is almost linear with a C—Ag—C angle of 178.60 (9) °. The two five membered rings are almost co-planar with C9 showing the maximum deviation from the least squares plane of 0.05 Å. The silver atom is 0.07 Å out of this plane and the C11–C16 and C28–C33 rings make angles of 46.61 ° and 41.77 ° repectively with it.

In the crystal there are C—H···F hydrogen bonds and π–π interactions which contribute to supermolecular structure. (Fig. 2) The π–π interactions are between rings N3/C25/N4/C26/C27 and N1/C8/N2/C9/C10 and rings C11–C16 and C28–C33 with the ring centroids being separated by 3.921 Å and 3.813 Å, respectively. [symmetry code: 1-*X*,1/2+Y,1/2-*Z* and 1-*X*,-1/2+Y,1/2-*Z*.] In addition C—H···π interactions involving the imidazole and benzene rings are also observed.

### Experimental

The title compound was prepared according to the reported procedures (Liu *et al.* 2003; Wang, Song *et al.* 2005). Colourless single crystals suitable for X-ray diffraction were obtained by recrystallization from dichloromethane and diethyl ether.

### Refinement

H atoms were placed in calculated positions with C—H = 0.95–0.99 Å, and refined in the riding mode with *U*_iso_(H) = 1.2*U*_eq_(C).

### Crystal data

**Table:**

[Ag(C_17_H_16_N_2_)_2_]PF_6_	*F*(000) = 1520
*M**_r_* = 749.48	*D*_x_ = 1.560 Mg m^−^^3^
Monoclinic, *P*2_1_/*c*	Mo *K*α radiation, λ = 0.71073 Å
*a* = 9.692 (2) Å	Cell parameters from 11560 reflections
*b* = 16.312 (4) Å	θ = 1.6–27.9°
*c* = 20.227 (5) Å	µ = 0.75 mm^−^^1^
β = 93.469 (3)°	*T* = 113 K
*V* = 3192.1 (12) Å^3^	Prism, colorless
*Z* = 4	0.20 × 0.18 × 0.12 mm

### Data collection

**Table:**

Rigaku Saturn CCD area detector diffractometer	5636 independent reflections
Radiation source: rotating anode	4775 reflections with *I* > 2σ(*I*)
multilayer	*R*_int_ = 0.043
Detector resolution: 14.63 pixels mm^-1^	θ_max_ = 25.0°, θ_min_ = 1.6°
ω and φ scans	*h* = −11→7
Absorption correction: multi-scan (*CrystalClear*; Rigaku/MSC, 2004)	*k* = −19→19
*T*_min_ = 0.865, *T*_max_ = 0.916	*l* = −22→24
21677 measured reflections	

### Refinement

**Table:**

Refinement on *F*^2^	Primary atom site location: structure-invariant direct methods
Least-squares matrix: full	Secondary atom site location: difference Fourier map
*R*[*F*^2^ > 2σ(*F*^2^)] = 0.031	Hydrogen site location: inferred from neighbouring sites
*wR*(*F*^2^) = 0.081	H-atom parameters constrained
*S* = 1.01	*w* = 1/[σ^2^(*F*_o_^2^) + (0.0457*P*)^2^] where *P* = (*F*_o_^2^ + 2*F*_c_^2^)/3
5636 reflections	(Δ/σ)_max_ = 0.002
417 parameters	Δρ_max_ = 1.29 e Å^−^^3^
0 restraints	Δρ_min_ = −0.38 e Å^−^^3^

### Special details

**Table:**

Geometry. All e.s.d.'s (except the e.s.d. in the dihedral angle between two l.s. planes) are estimated using the full covariance matrix. The cell e.s.d.'s are taken into account individually in the estimation of e.s.d.'s in distances, angles and torsion angles; correlations between e.s.d.'s in cell parameters are only used when they are defined by crystal symmetry. An approximate (isotropic) treatment of cell e.s.d.'s is used for estimating e.s.d.'s involving l.s. planes.
Refinement. Refinement of *F*^2^ against ALL reflections. The weighted *R*-factor *wR* and goodness of fit *S* are based on *F*^2^, conventional *R*-factors *R* are based on *F*, with *F* set to zero for negative *F*^2^. The threshold expression of *F*^2^ > σ(*F*^2^) is used only for calculating *R*-factors(gt) *etc*. and is not relevant to the choice of reflections for refinement. *R*-factors based on *F*^2^ are statistically about twice as large as those based on *F*, and *R*- factors based on ALL data will be even larger.

### Fractional atomic coordinates and isotropic or equivalent isotropic displacement parameters (Å^2^)

**Table:**

	*x*	*y*	*z*	*U*_iso_*/*U*_eq_	
Ag1	0.41572 (2)	0.859060 (11)	0.816377 (9)	0.02055 (8)	
N1	0.2579 (2)	1.00527 (12)	0.74697 (10)	0.0201 (5)	
N2	0.4069 (2)	1.04857 (12)	0.82125 (10)	0.0201 (5)	
N3	0.5671 (2)	0.71360 (12)	0.88999 (11)	0.0218 (5)	
N4	0.4187 (2)	0.66895 (13)	0.81610 (10)	0.0203 (5)	
C1	0.3421 (3)	0.91678 (15)	0.61584 (14)	0.0236 (6)	
H1	0.3971	0.8891	0.6493	0.028*	
C2	0.3847 (3)	0.91908 (15)	0.55177 (14)	0.0269 (6)	
H2	0.4678	0.8925	0.5413	0.032*	
C3	0.3058 (3)	0.96022 (16)	0.50299 (14)	0.0282 (7)	
H3	0.3345	0.9619	0.4589	0.034*	
C4	0.1846 (3)	0.99894 (16)	0.51871 (13)	0.0273 (6)	
H4	0.1307	1.0277	0.4855	0.033*	
C5	0.1421 (3)	0.99574 (16)	0.58290 (13)	0.0237 (6)	
H5	0.0587	1.0222	0.5933	0.028*	
C6	0.2194 (3)	0.95459 (14)	0.63189 (13)	0.0211 (6)	
C7	0.1735 (3)	0.95170 (15)	0.70193 (13)	0.0240 (6)	
H7A	0.0756	0.9689	0.7019	0.029*	
H7B	0.1800	0.8946	0.7184	0.029*	
C8	0.3559 (3)	0.97862 (15)	0.79212 (13)	0.0194 (6)	
C9	0.3392 (3)	1.11697 (15)	0.79473 (13)	0.0230 (6)	
H9	0.3556	1.1724	0.8074	0.028*	
C10	0.2466 (3)	1.09047 (15)	0.74812 (14)	0.0248 (6)	
H10	0.1851	1.1232	0.7210	0.030*	
C11	0.5114 (3)	1.05048 (15)	0.87445 (13)	0.0215 (6)	
C12	0.4931 (3)	1.10008 (17)	0.92862 (14)	0.0296 (7)	
H12	0.4131	1.1335	0.9302	0.035*	
C13	0.5936 (3)	1.10046 (17)	0.98088 (14)	0.0320 (7)	
H13	0.5819	1.1353	1.0178	0.038*	
C14	0.7096 (3)	1.05160 (17)	0.98046 (13)	0.0279 (6)	
C15	0.7260 (3)	1.00243 (16)	0.92519 (13)	0.0257 (6)	
H15	0.8052	0.9683	0.9240	0.031*	
C16	0.6293 (3)	1.00224 (15)	0.87199 (13)	0.0232 (6)	
H16	0.6433	0.9695	0.8342	0.028*	
C17	0.8157 (3)	1.0520 (2)	1.03823 (15)	0.0394 (8)	
H17A	0.8500	1.1080	1.0458	0.059*	
H17B	0.8929	1.0161	1.0284	0.059*	
H17C	0.7732	1.0323	1.0780	0.059*	
C18	0.8641 (3)	0.77442 (17)	0.86335 (14)	0.0329 (7)	
H18	0.8072	0.7999	0.8294	0.040*	
C19	1.0014 (3)	0.7597 (2)	0.85337 (16)	0.0423 (8)	
H19	1.0380	0.7742	0.8125	0.051*	
C20	1.0861 (3)	0.72373 (19)	0.90282 (17)	0.0424 (8)	
H20	1.1810	0.7142	0.8962	0.051*	
C21	1.0310 (3)	0.70167 (18)	0.96223 (16)	0.0376 (8)	
H21	1.0882	0.6769	0.9964	0.045*	
C22	0.8923 (3)	0.71589 (16)	0.97149 (14)	0.0284 (7)	
H22	0.8548	0.7004	1.0120	0.034*	
C23	0.8082 (3)	0.75253 (15)	0.92215 (13)	0.0232 (6)	
C24	0.6570 (3)	0.76661 (15)	0.93234 (13)	0.0261 (6)	
H24A	0.6395	0.7561	0.9793	0.031*	
H24B	0.6338	0.8246	0.9226	0.031*	
C25	0.4730 (3)	0.73940 (15)	0.84277 (13)	0.0219 (6)	
C26	0.4790 (3)	0.60099 (15)	0.84766 (13)	0.0239 (6)	
H26	0.4582	0.5451	0.8382	0.029*	
C27	0.5712 (3)	0.62885 (15)	0.89357 (14)	0.0250 (6)	
H27	0.6288	0.5968	0.9231	0.030*	
C28	0.3152 (3)	0.66399 (15)	0.76335 (13)	0.0214 (6)	
C29	0.3165 (3)	0.71662 (15)	0.70946 (13)	0.0243 (6)	
H29	0.3885	0.7558	0.7067	0.029*	
C30	0.2120 (3)	0.71147 (15)	0.65974 (14)	0.0284 (7)	
H30	0.2116	0.7487	0.6236	0.034*	
C31	0.1089 (3)	0.65370 (15)	0.66140 (14)	0.0256 (6)	
C32	0.1107 (3)	0.59955 (16)	0.71508 (14)	0.0259 (6)	
H32	0.0413	0.5586	0.7165	0.031*	
C33	0.2115 (3)	0.60464 (15)	0.76600 (13)	0.0229 (6)	
H33	0.2105	0.5682	0.8026	0.027*	
C34	−0.0037 (3)	0.64745 (17)	0.60683 (15)	0.0359 (7)	
H34A	−0.0646	0.6953	0.6083	0.054*	
H34B	−0.0576	0.5974	0.6128	0.054*	
H34C	0.0378	0.6455	0.5639	0.054*	
P1	0.76650 (7)	0.85484 (4)	0.67062 (3)	0.02175 (17)	
F1	0.84018 (19)	0.77564 (9)	0.70222 (8)	0.0389 (4)	
F2	0.89576 (18)	0.86984 (11)	0.62716 (9)	0.0443 (5)	
F3	0.69599 (18)	0.79928 (10)	0.61225 (8)	0.0374 (4)	
F4	0.63656 (18)	0.83735 (13)	0.71351 (9)	0.0523 (5)	
F5	0.6920 (2)	0.93310 (10)	0.63843 (9)	0.0517 (5)	
F6	0.83605 (18)	0.90927 (9)	0.72922 (8)	0.0357 (4)	

### Atomic displacement parameters (Å^2^)

**Table:**

	*U*^11^	*U*^22^	*U*^33^	*U*^12^	*U*^13^	*U*^23^
Ag1	0.02564 (13)	0.01226 (11)	0.02389 (13)	0.00213 (8)	0.00264 (9)	0.00104 (8)
N1	0.0228 (12)	0.0150 (10)	0.0228 (12)	0.0007 (10)	0.0041 (10)	−0.0013 (9)
N2	0.0236 (12)	0.0145 (11)	0.0228 (12)	0.0016 (9)	0.0050 (10)	0.0008 (9)
N3	0.0234 (12)	0.0171 (11)	0.0249 (13)	0.0034 (10)	0.0022 (10)	0.0021 (9)
N4	0.0227 (12)	0.0136 (10)	0.0248 (13)	0.0027 (9)	0.0038 (10)	0.0031 (9)
C1	0.0221 (14)	0.0163 (12)	0.0320 (16)	−0.0006 (12)	−0.0016 (12)	0.0035 (11)
C2	0.0247 (15)	0.0221 (14)	0.0346 (17)	−0.0020 (12)	0.0063 (13)	−0.0044 (12)
C3	0.0301 (16)	0.0279 (15)	0.0268 (16)	−0.0100 (13)	0.0018 (13)	−0.0051 (12)
C4	0.0291 (16)	0.0268 (14)	0.0248 (16)	−0.0093 (13)	−0.0088 (13)	0.0009 (12)
C5	0.0168 (14)	0.0222 (13)	0.0315 (16)	−0.0028 (12)	−0.0027 (12)	−0.0027 (12)
C6	0.0206 (14)	0.0147 (12)	0.0278 (15)	−0.0066 (11)	0.0007 (12)	−0.0017 (11)
C7	0.0215 (14)	0.0193 (13)	0.0310 (16)	−0.0040 (12)	0.0009 (12)	0.0013 (11)
C8	0.0198 (14)	0.0186 (13)	0.0204 (14)	−0.0005 (11)	0.0048 (12)	−0.0002 (11)
C9	0.0262 (15)	0.0122 (12)	0.0314 (16)	0.0034 (12)	0.0066 (13)	0.0013 (11)
C10	0.0231 (15)	0.0168 (13)	0.0349 (17)	0.0038 (12)	0.0057 (13)	0.0049 (11)
C11	0.0256 (15)	0.0175 (13)	0.0219 (14)	−0.0019 (12)	0.0045 (12)	0.0015 (11)
C12	0.0301 (16)	0.0281 (15)	0.0308 (17)	0.0078 (13)	0.0041 (14)	−0.0043 (12)
C13	0.0380 (18)	0.0334 (16)	0.0250 (16)	0.0053 (14)	0.0048 (14)	−0.0066 (13)
C14	0.0288 (16)	0.0313 (15)	0.0239 (15)	−0.0017 (13)	0.0029 (12)	0.0043 (12)
C15	0.0218 (15)	0.0225 (14)	0.0333 (16)	0.0021 (12)	0.0067 (13)	0.0036 (12)
C16	0.0257 (15)	0.0176 (13)	0.0270 (15)	−0.0005 (12)	0.0073 (12)	−0.0030 (11)
C17	0.0378 (19)	0.0473 (19)	0.0327 (18)	0.0019 (16)	−0.0003 (15)	0.0002 (14)
C18	0.0410 (19)	0.0330 (16)	0.0245 (16)	−0.0019 (15)	0.0001 (14)	0.0027 (12)
C19	0.042 (2)	0.051 (2)	0.0350 (19)	−0.0101 (17)	0.0121 (16)	−0.0057 (15)
C20	0.0267 (17)	0.047 (2)	0.055 (2)	−0.0058 (15)	0.0118 (16)	−0.0201 (17)
C21	0.0300 (17)	0.0363 (17)	0.045 (2)	0.0046 (15)	−0.0115 (15)	−0.0100 (14)
C22	0.0349 (17)	0.0234 (14)	0.0265 (16)	−0.0014 (13)	−0.0008 (13)	−0.0017 (12)
C23	0.0285 (15)	0.0143 (12)	0.0264 (15)	−0.0014 (12)	−0.0007 (12)	−0.0053 (11)
C24	0.0328 (16)	0.0218 (14)	0.0232 (15)	0.0033 (13)	−0.0010 (13)	−0.0025 (11)
C25	0.0229 (15)	0.0191 (13)	0.0240 (15)	0.0027 (12)	0.0039 (12)	0.0001 (11)
C26	0.0263 (15)	0.0154 (13)	0.0305 (16)	0.0058 (12)	0.0067 (13)	0.0066 (11)
C27	0.0261 (15)	0.0186 (13)	0.0307 (16)	0.0067 (12)	0.0047 (13)	0.0050 (11)
C28	0.0234 (15)	0.0153 (12)	0.0259 (15)	0.0060 (12)	0.0045 (12)	−0.0019 (11)
C29	0.0320 (16)	0.0148 (12)	0.0263 (15)	−0.0015 (12)	0.0043 (13)	0.0002 (11)
C30	0.0417 (18)	0.0171 (13)	0.0263 (16)	0.0061 (13)	0.0015 (14)	0.0036 (11)
C31	0.0282 (16)	0.0225 (14)	0.0260 (15)	0.0076 (13)	0.0000 (12)	−0.0057 (11)
C32	0.0266 (15)	0.0195 (14)	0.0321 (16)	0.0012 (12)	0.0064 (13)	−0.0025 (12)
C33	0.0257 (15)	0.0186 (13)	0.0251 (15)	0.0014 (12)	0.0077 (12)	0.0016 (11)
C34	0.0393 (19)	0.0317 (16)	0.0362 (19)	0.0047 (14)	−0.0022 (15)	−0.0054 (13)
P1	0.0225 (4)	0.0188 (3)	0.0241 (4)	−0.0008 (3)	0.0027 (3)	0.0009 (3)
F1	0.0516 (11)	0.0235 (8)	0.0403 (10)	0.0065 (8)	−0.0081 (9)	0.0006 (7)
F2	0.0345 (10)	0.0628 (12)	0.0370 (11)	−0.0186 (9)	0.0119 (8)	−0.0025 (9)
F3	0.0408 (10)	0.0368 (9)	0.0336 (10)	−0.0077 (8)	−0.0071 (8)	−0.0050 (7)
F4	0.0329 (11)	0.0815 (14)	0.0443 (12)	−0.0074 (10)	0.0168 (9)	−0.0011 (10)
F5	0.0726 (14)	0.0287 (9)	0.0514 (12)	0.0177 (10)	−0.0154 (10)	0.0024 (8)
F6	0.0487 (11)	0.0246 (8)	0.0330 (10)	−0.0020 (8)	−0.0046 (8)	−0.0071 (7)

### Geometric parameters (Å, °)

**Table:**

Ag1—C8	2.085 (2)	C16—H16	0.9500
Ag1—C25	2.090 (2)	C17—H17A	0.9800
N1—C8	1.349 (3)	C17—H17B	0.9800
N1—C10	1.395 (3)	C17—H17C	0.9800
N1—C7	1.474 (3)	C18—C19	1.380 (4)
N2—C8	1.363 (3)	C18—C23	1.383 (4)
N2—C9	1.386 (3)	C18—H18	0.9500
N2—C11	1.433 (3)	C19—C20	1.385 (4)
N3—C25	1.348 (3)	C19—H19	0.9500
N3—C27	1.385 (3)	C20—C21	1.392 (4)
N3—C24	1.466 (3)	C20—H20	0.9500
N4—C25	1.362 (3)	C21—C22	1.387 (4)
N4—C26	1.390 (3)	C21—H21	0.9500
N4—C28	1.422 (3)	C22—C23	1.385 (4)
C1—C2	1.384 (4)	C22—H22	0.9500
C1—C6	1.395 (4)	C23—C24	1.510 (4)
C1—H1	0.9500	C24—H24A	0.9900
C2—C3	1.385 (4)	C24—H24B	0.9900
C2—H2	0.9500	C26—C27	1.329 (4)
C3—C4	1.387 (4)	C26—H26	0.9500
C3—H3	0.9500	C27—H27	0.9500
C4—C5	1.387 (4)	C28—C29	1.388 (4)
C4—H4	0.9500	C28—C33	1.399 (4)
C5—C6	1.380 (4)	C29—C30	1.386 (4)
C5—H5	0.9500	C29—H29	0.9500
C6—C7	1.511 (3)	C30—C31	1.376 (4)
C7—H7A	0.9900	C30—H30	0.9500
C7—H7B	0.9900	C31—C32	1.399 (4)
C9—C10	1.333 (4)	C31—C34	1.508 (4)
C9—H9	0.9500	C32—C33	1.378 (4)
C10—H10	0.9500	C32—H32	0.9500
C11—C12	1.382 (4)	C33—H33	0.9500
C11—C16	1.391 (4)	C34—H34A	0.9800
C12—C13	1.393 (4)	C34—H34B	0.9800
C12—H12	0.9500	C34—H34C	0.9800
C13—C14	1.379 (4)	P1—F5	1.5867 (17)
C13—H13	0.9500	P1—F1	1.5913 (17)
C14—C15	1.393 (4)	P1—F2	1.5923 (18)
C14—C17	1.509 (4)	P1—F6	1.5971 (16)
C15—C16	1.384 (4)	P1—F4	1.5977 (18)
C15—H15	0.9500	P1—F3	1.6077 (17)
			
C8—Ag1—C25	178.60 (9)	C19—C18—H18	119.6
C8—N1—C10	111.3 (2)	C23—C18—H18	119.6
C8—N1—C7	124.7 (2)	C18—C19—C20	120.2 (3)
C10—N1—C7	124.1 (2)	C18—C19—H19	119.9
C8—N2—C9	110.8 (2)	C20—C19—H19	119.9
C8—N2—C11	124.4 (2)	C19—C20—C21	119.4 (3)
C9—N2—C11	124.7 (2)	C19—C20—H20	120.3
C25—N3—C27	111.4 (2)	C21—C20—H20	120.3
C25—N3—C24	125.6 (2)	C22—C21—C20	119.9 (3)
C27—N3—C24	123.0 (2)	C22—C21—H21	120.0
C25—N4—C26	110.5 (2)	C20—C21—H21	120.0
C25—N4—C28	125.7 (2)	C23—C22—C21	120.5 (3)
C26—N4—C28	123.9 (2)	C23—C22—H22	119.8
C2—C1—C6	120.8 (2)	C21—C22—H22	119.8
C2—C1—H1	119.6	C18—C23—C22	119.1 (3)
C6—C1—H1	119.6	C18—C23—C24	120.8 (2)
C1—C2—C3	119.8 (3)	C22—C23—C24	120.1 (2)
C1—C2—H2	120.1	N3—C24—C23	112.2 (2)
C3—C2—H2	120.1	N3—C24—H24A	109.2
C2—C3—C4	119.7 (3)	C23—C24—H24A	109.2
C2—C3—H3	120.1	N3—C24—H24B	109.2
C4—C3—H3	120.1	C23—C24—H24B	109.2
C5—C4—C3	120.1 (3)	H24A—C24—H24B	107.9
C5—C4—H4	120.0	N3—C25—N4	104.2 (2)
C3—C4—H4	120.0	N3—C25—Ag1	129.10 (18)
C6—C5—C4	120.7 (3)	N4—C25—Ag1	126.64 (19)
C6—C5—H5	119.6	C27—C26—N4	107.1 (2)
C4—C5—H5	119.6	C27—C26—H26	126.4
C5—C6—C1	118.8 (2)	N4—C26—H26	126.4
C5—C6—C7	120.7 (2)	C26—C27—N3	106.8 (2)
C1—C6—C7	120.5 (2)	C26—C27—H27	126.6
N1—C7—C6	112.2 (2)	N3—C27—H27	126.6
N1—C7—H7A	109.2	C29—C28—C33	119.9 (3)
C6—C7—H7A	109.2	C29—C28—N4	120.9 (2)
N1—C7—H7B	109.2	C33—C28—N4	119.1 (2)
C6—C7—H7B	109.2	C30—C29—C28	119.4 (3)
H7A—C7—H7B	107.9	C30—C29—H29	120.3
N1—C8—N2	104.2 (2)	C28—C29—H29	120.3
N1—C8—Ag1	129.43 (18)	C31—C30—C29	121.6 (3)
N2—C8—Ag1	126.36 (19)	C31—C30—H30	119.2
C10—C9—N2	107.2 (2)	C29—C30—H30	119.2
C10—C9—H9	126.4	C30—C31—C32	118.5 (3)
N2—C9—H9	126.4	C30—C31—C34	121.6 (3)
C9—C10—N1	106.5 (2)	C32—C31—C34	120.0 (3)
C9—C10—H10	126.7	C33—C32—C31	121.1 (3)
N1—C10—H10	126.7	C33—C32—H32	119.4
C12—C11—C16	120.3 (3)	C31—C32—H32	119.4
C12—C11—N2	119.3 (2)	C32—C33—C28	119.4 (2)
C16—C11—N2	120.4 (2)	C32—C33—H33	120.3
C11—C12—C13	119.2 (3)	C28—C33—H33	120.3
C11—C12—H12	120.4	C31—C34—H34A	109.5
C13—C12—H12	120.4	C31—C34—H34B	109.5
C14—C13—C12	121.7 (3)	H34A—C34—H34B	109.5
C14—C13—H13	119.1	C31—C34—H34C	109.5
C12—C13—H13	119.1	H34A—C34—H34C	109.5
C13—C14—C15	118.0 (3)	H34B—C34—H34C	109.5
C13—C14—C17	120.7 (3)	F5—P1—F1	179.27 (11)
C15—C14—C17	121.3 (3)	F5—P1—F2	90.20 (11)
C16—C15—C14	121.4 (3)	F1—P1—F2	89.85 (10)
C16—C15—H15	119.3	F5—P1—F6	91.05 (9)
C14—C15—H15	119.3	F1—P1—F6	89.67 (9)
C15—C16—C11	119.4 (2)	F2—P1—F6	90.77 (10)
C15—C16—H16	120.3	F5—P1—F4	90.71 (11)
C11—C16—H16	120.3	F1—P1—F4	89.21 (11)
C14—C17—H17A	109.5	F2—P1—F4	178.48 (11)
C14—C17—H17B	109.5	F6—P1—F4	90.43 (10)
H17A—C17—H17B	109.5	F5—P1—F3	89.41 (10)
C14—C17—H17C	109.5	F1—P1—F3	89.87 (9)
H17A—C17—H17C	109.5	F2—P1—F3	89.71 (10)
H17B—C17—H17C	109.5	F6—P1—F3	179.34 (10)
C19—C18—C23	120.8 (3)	F4—P1—F3	89.09 (10)
			
C6—C1—C2—C3	0.8 (4)	C23—C18—C19—C20	−1.0 (5)
C1—C2—C3—C4	0.1 (4)	C18—C19—C20—C21	0.8 (5)
C2—C3—C4—C5	−0.6 (4)	C19—C20—C21—C22	−0.1 (4)
C3—C4—C5—C6	0.3 (4)	C20—C21—C22—C23	−0.5 (4)
C4—C5—C6—C1	0.6 (4)	C19—C18—C23—C22	0.4 (4)
C4—C5—C6—C7	179.5 (2)	C19—C18—C23—C24	−178.3 (3)
C2—C1—C6—C5	−1.1 (4)	C21—C22—C23—C18	0.3 (4)
C2—C1—C6—C7	179.9 (2)	C21—C22—C23—C24	179.1 (2)
C8—N1—C7—C6	−104.8 (3)	C25—N3—C24—C23	−117.8 (3)
C10—N1—C7—C6	75.0 (3)	C27—N3—C24—C23	61.1 (3)
C5—C6—C7—N1	−106.0 (3)	C18—C23—C24—N3	68.3 (3)
C1—C6—C7—N1	72.9 (3)	C22—C23—C24—N3	−110.4 (3)
C10—N1—C8—N2	−0.5 (3)	C27—N3—C25—N4	−0.6 (3)
C7—N1—C8—N2	179.3 (2)	C24—N3—C25—N4	178.4 (2)
C10—N1—C8—Ag1	177.56 (18)	C27—N3—C25—Ag1	177.84 (19)
C7—N1—C8—Ag1	−2.6 (4)	C24—N3—C25—Ag1	−3.1 (4)
C9—N2—C8—N1	0.9 (3)	C26—N4—C25—N3	0.6 (3)
C11—N2—C8—N1	177.8 (2)	C28—N4—C25—N3	−179.3 (2)
C9—N2—C8—Ag1	−177.31 (17)	C26—N4—C25—Ag1	−177.89 (17)
C11—N2—C8—Ag1	−0.4 (3)	C28—N4—C25—Ag1	2.2 (4)
C25—Ag1—C8—N1	−103 (4)	C8—Ag1—C25—N3	−75 (4)
C25—Ag1—C8—N2	75 (4)	C8—Ag1—C25—N4	103 (4)
C8—N2—C9—C10	−0.9 (3)	C25—N4—C26—C27	−0.4 (3)
C11—N2—C9—C10	−177.8 (2)	C28—N4—C26—C27	179.5 (2)
N2—C9—C10—N1	0.5 (3)	N4—C26—C27—N3	0.0 (3)
C8—N1—C10—C9	0.0 (3)	C25—N3—C27—C26	0.4 (3)
C7—N1—C10—C9	−179.8 (2)	C24—N3—C27—C26	−178.7 (2)
C8—N2—C11—C12	−133.5 (3)	C25—N4—C28—C29	40.1 (4)
C9—N2—C11—C12	43.0 (4)	C26—N4—C28—C29	−139.7 (3)
C8—N2—C11—C16	45.7 (3)	C25—N4—C28—C33	−140.4 (3)
C9—N2—C11—C16	−137.7 (3)	C26—N4—C28—C33	39.8 (3)
C16—C11—C12—C13	−0.6 (4)	C33—C28—C29—C30	2.1 (4)
N2—C11—C12—C13	178.7 (2)	N4—C28—C29—C30	−178.4 (2)
C11—C12—C13—C14	−1.1 (4)	C28—C29—C30—C31	−2.0 (4)
C12—C13—C14—C15	1.4 (4)	C29—C30—C31—C32	0.3 (4)
C12—C13—C14—C17	−178.9 (3)	C29—C30—C31—C34	−179.2 (3)
C13—C14—C15—C16	0.1 (4)	C30—C31—C32—C33	1.3 (4)
C17—C14—C15—C16	−179.7 (3)	C34—C31—C32—C33	−179.2 (2)
C14—C15—C16—C11	−1.7 (4)	C31—C32—C33—C28	−1.2 (4)
C12—C11—C16—C15	2.0 (4)	C29—C28—C33—C32	−0.6 (4)
N2—C11—C16—C15	−177.3 (2)	N4—C28—C33—C32	179.9 (2)

### Hydrogen-bond geometry (Å, °)

**Table:**

Cg3 is the centroid of the C1–C6 ring.

**Table:**

*D*—H···*A*	*D*—H	H···*A*	*D*···*A*	*D*—H···*A*
C9—H9···F1^i^	0.95	2.54	3.120 (6)	119
C26—H26···F5^ii^	0.95	2.40	3.222 (1)	144
C34—H34A···F1^iii^	0.98	2.53	3.276 (8)	133
C27—H27···Cg3^ii^	0.95	2.50	3.295 (1)	140

Symmetry codes: (i) −*x*+1, *y*−1/2, −*z*+1/2; (ii) −*x*+1, *y*+1/2, −*z*+1/2; (iii) *x*+1, *y*, *z*.
